# Retraction Note: Endothelial progenitor cell-derived exosomes, loaded with miR-126, promoted deep vein thrombosis resolution and recanalization

**DOI:** 10.1186/s13287-019-1264-3

**Published:** 2019-06-11

**Authors:** Jiacheng Sun, Zhiwei Zhang, Teng Ma, Ziying Yang, Jinlong Zhang, Xuan Liu, Da Lu, Zhenya Shen, Junjie Yang, Qingyou Meng

**Affiliations:** 1 Department of Vascular Surgery, The Second Affiliated Hospital of Soochow University, Suzhou, 215000 China; 2 Department of Biomedical Engineering, University of Alabama at Birmingham, Birmingham, Alabama 35294 USA; 3 Department of Cardiothoracic Surgery, The Second Affiliated Hospital of Soochow University, Suzhou, 215004 China; 4 Department of Cardiovascular Surgery of the First Affiliated Hospital and Institute for Cardiovascular Science, Soochow University, Suzhou, 215000 China

**Retraction Note: Stem Cell Res Ther (2018) 9:223**

**https://doi.org/10.1186/s13287-018-0952-8**

The authors have retracted this article [1] because there is erroneous data in Figure 1C. Flow cytometry results of EPC cell markers CD31 and CD45 could not be replicated. Due to incorrect cell markers, the cells cultured may not have been pure EPCs. Therefore the scientific content of the article is no longer reliable. All authors agree to this retraction.
